# Isolated pericardial cystic Echinococcosis: A rare clinical presentation

**DOI:** 10.12669/pjms.38.3.4965

**Published:** 2022

**Authors:** Pratikshya Thapaliya, Taimur Asif Ali, Mahwish Mahboob Bhutta

**Affiliations:** 1 Pratikshya Thapaliya, FCPS-II Postgraduate Trainee Department of Cardiovascular surgery, National Institute of Cardiovascular Diseases, Karachi, Pakistan; 2 Taimur Asif Ali Consultant Surgeon, Department of Cardiovascular surgery, National Institute of Cardiovascular Diseases, Karachi, Pakistan; 3 Mahwish Mahboob Bhutta FCPS-II post graduate trainee, PNS SHIFA Hospital, Karachi, Pakistan

**Keywords:** Cystic Echinococcosis, Pericarditis, Pericardial Cyst

## Abstract

Isolated pericardial Hydatid cyst without involvement of other viscera is a rare condition with reported incidence of 0.5-2% of all cases of cystic echinococcosis even in the countries endemic for the disease. Hydatid disease is a major public health concern in the animal raising regions worldwide. Pericardial hydatid disease can be asymptomatic or present with varying symptoms from atypical chest pain, arrhythmias, rupture and tamponade to anaphylaxis. Early diagnosis and surgical treatment is necessary to prevent fatal complications. Here we report a case of symptomatic isolated pericardial hydatid cyst who presented with epigastric pain.

## INTRODUCTION

Echinococcosis is a zoonotic infection, caused by larvae of tapeworm Echinococcus granulosus.^1^ It is endemic in many animal raising areas worldwide and poses a public health concern. WHO included cystic echinococcosis in a subgroup of selected neglected tropic diseases (NTDs) to be addressed within its 2008-2015 strategic plans to control NTDs.^2^ In the parasite’s life cycle, the primary host is generally a canine which releases ova in its feces, the intermediate hosts (sheep, cattle, horses, goats, pigs) then ingest the infected grass or vegetables or drink infected water and become infested? Embryos are released in the intestine of intermediate host and penetrate the duodenal or jejunal wall from where they enter the portal circulation.^1^ Liver is the commonly affected organ in hydatid disease (60-70%), however some embroyes may also bypass the liver and enter right heart through the hepatic vein and inferior vena cava and into lungs which is the second most common organ to be infected (10-15%).^3^ Other embryos may enter the systemic circulation and disseminate to other organs. Another pathway to the lungs is through the intestinal lymphatics into the thoracic duct and then to the right heart, finally reaching the lung.^1^ Humans are the accidental hosts infected by ingesting contaminated water or food or by contact directly with infected dogs.^1^ Cardiac involvement by hydatid cysts presents only in 0.5-2% of cases of systemic echinococcosis.^3^-^5^ Isolated pericardial cyst without myocardial involvement is very rare. High degree of suspicion is needed for cardiac cystic disease for early diagnosis.

## CASE PRESENTATION

**Fig.1:**
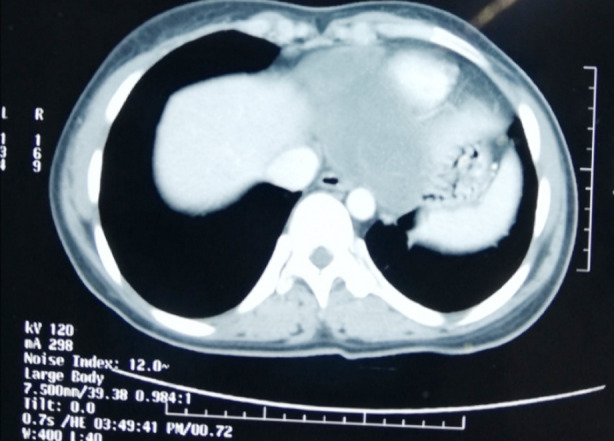
Hypodense lesion along the inferior cardiac margin with linear hyperdensities within the lesion.

**Fig.2:**
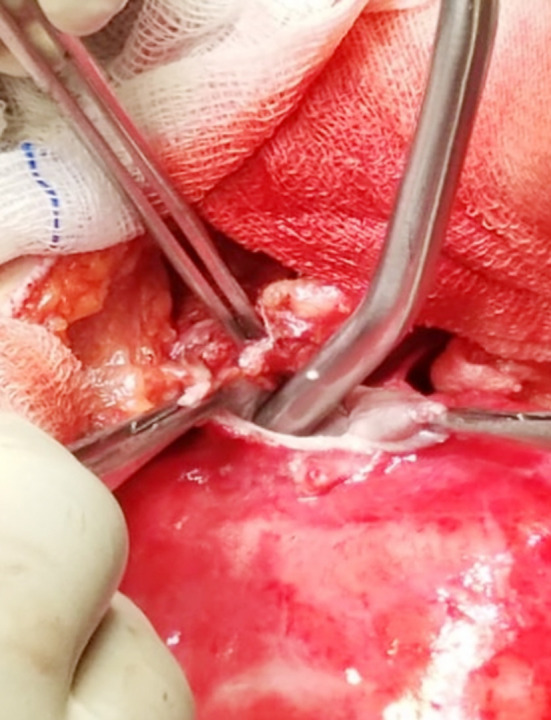
Cystostomy and suctioning of hydatid fluid after packing the surrounding space with 10% saline soaked gauze.

We present a case of a 20 years female resident of Afghanistan, involved in animal farming and with regular close contact with sheep, cows, camel and dogs, presented to us with complaint of epigastric and left upper abdominal pain from 10 months. The pain was dull, aching in character, referring to tip of her left shoulder. Her father was operated few months ago for liver hydatid. On physical examination, her temperature was 37°C, pulse rate of 100 beats/min and BP of 110/60 mmHg. Systemic examination was unremarkable. ECG showed normal sinus rhythm. Chest x-ray showed abnormal cardiac silhouette on left side. Transthoracic echocardiography revealed echo-dense space located antero-inferiorly in pericardium. CT chest and abdomen with contrast demonstrated hypodense lesion of fluid attenuation, along the inferior cardiac margin with multiple linear hyper densities within it (Fig.1). No other lesion in chest or abdomen. Echinococcal antibody via ELISA was positive. Given the findings pericardial hydatid cyst was diagnosed. Patient was started on albendazole one week prior to surgery and was operated via median sternotomy. Dense pericardial adhesions with features of pericarditis were noted. Pus filled hydatid cyst was found with no communication with pleura or peritoneum correlating with the findings on CT scan. Pericardium was packed with 10% saline before cystostomy. Suction and instillation of 10%saline into the cyst followed by removal of germinal membrane performed (Fig.2). Pericyst left intact over the epicardium and diaphragmatic surface and pericardial cavity washed with hypertonic saline. Post-operative course remained uneventful, and patient was discharged on 5^th^ post-operative day with albendazole 10mg/kg/day with two weekly cycles for three months.

## DISCUSSION

In cardiac hydatid cyst, embryos reach the right atrium from intestine through the liver and azygos vein, access into the left ventricle and coronary arteries or via rupture of a lung cyst into the pulmonary vein.^6^,^7^ The anatomical site of location in the heart depends on the amount of regional vascularization.^6^ The most frequent location of the cyst is the myocardium, particularly the left ventricular free wall (75%),right ventricle(15%) and interventricular septum(5-9%),left atrium (8%),pericardium(8%), right atrium(3-4%).^5^ In our case, the hydatid cyst was located within the pericardial cavity without myocardial involvement.

Clinical presentation depends on size, location, number of cysts and presence of complications.^5^,^8^ It can remain asymptomatic or can have symptoms like atypical chest pain, dyspnea and orthopnea due to compressive effects. Cardiac hydatidosis can prove to be fatal due to complications like cyst rupture leading to tamponade, anaphylactic shock, systemic or pulmonary hydatid embolization, valve obstruction or regurgitation due to papillary muscle involvement, arrhythmia and atrioventricular conduction defect, pericarditis with pericardial effusion.^3^-^5^

Early diagnosis and urgent treatment are needed even in asymptomatic individuals. Hydatid cyst should be considered in differential diagnosis for any intracardiac cystic mass. Chest x-ray may show abnormal cardiac silhouette.^5^ Echocardiography is the investigation of choice. Transthoracic echocardiography is safe and helps in characterizing the lesion. Gradient echo technique provides morphological information about cardiac cysts along with effect of cysts on cardiac function.^5^ An assessment of the heart and main vessels by echocardiography may be recommended for all individuals diagnosed with CE, even if they are asymptomatic.^5^,^8^ CT/MRI is effective for confirming diagnosis and looking for lesions in other locations. It can show the site of the abnormality and provide precise information for the surgeon.^9^ Indirect hemagglutination and ELISA tests are sensitive for hepatic cases (85–98%), less sensitive for lung involvement (50–56%), and poorly sensitive for another organ involvement (25–56%).^10^

The patient presented in this case had bacterial superinfection of hydatid cysts. Previous reports have shown a rate of superinfection in cystic hydatidosis of 1 – 8 % in newly diagnosed cases.^4^,^5^ Super infection might change the echogenicity of cyst as was noted in our case.

Once diagnosis is established surgery is the best management option with pre and post operative chemotherapy using albendazole.^1^,^4^,^5^,^11^ Intraoperative preventive measures are needed to prevent dissemination and recurrence. In our case we used 10% saline as scolicidal agent.

## CONCLUSION

Cardiac hydatidosis is rare but should be suspected in any case presenting with cystic cardiac or pericardial mass, especially if there is history of travel to endemic area or contact with animals. Early diagnosis leads to early treatment and helps in preventing the grave complications of disease progression. Awareness of the disease and prophylaxis is needed in the endemic regions for primary prevention of the disease.

### Authors Contribution:

**PT:** Conception, design, literature search, analysis, drafting the work, agreement to be accountable for all aspects of the work.

**TAA:** Conception, critical revision of work, final approval of version to be published, agreement to be accountable for all aspects of the work.

**MMB:** Literature search, analysis, drafting the work, agreement to be accountable for all aspects of the work.
